# Remodelling sympathetic innervation in rat pancreatic islets ontogeny

**DOI:** 10.1186/1471-213X-9-34

**Published:** 2009-06-17

**Authors:** Siraam Cabrera-Vásquez, Víctor Navarro-Tableros, Carmen Sánchez-Soto, Gabriel Gutiérrez-Ospina, Marcia Hiriart

**Affiliations:** 1 Biophysics Department, Instituto de Fisiología Celular – Neuroscience, Universidad Nacional Autónoma de México, Mexico DF, 04510, México; 2 Cell Biology and Physiology Department, Instituto de Investigaciones Biomédicas, Universidad Nacional Autónoma de México, Mexico DF, 04510, México

## Abstract

**Background:**

Pancreatic islets are not fully developed at birth and it is not clear how they are vascularised and innervated. Nerve Growth Factor (NGF) is required to guide sympathetic neurons that innervate peripheral organs and also in cardiovascular system and ovary angiogenesis. Pancreatic beta cells of a transgenic mouse that over-expressed NGF in attracts sympathetic hyper-innervation towards them. Moreover, we have previously demonstrated that adult beta cells synthesize and secrete NGF; however, we do not know how is NGF secreted during development, nor if it might be trophic for sympathetic innervation and survival in the pancreas.

We analyzed sympathetic innervation and vasculature development in rat pancreatic islets at different developmental stages; foetal (F19), early postnatal (P1), weaning period (P20) and adults. We temporarily correlated these events to NGF secretion by islet cells.

**Results:**

Sympathetic fibres reached pancreatic islets in the early postnatal period, apparently following blood vessels. The maximal number of sympathetic fibres (TH immunopositive) in the periphery of the islets was observed at P20, and then fibres entered the islets and reached the core where beta cells are mainly located. The number of fibres decreased from that stage to adulthood. At all stages studied, islet cells secreted NGF and also expressed the high affinity receptor TrkA. Foetal and neonatal isolated islet cells secreted more NGF than adults. TrkA receptors were expressed at all stages in pancreatic sympathetic fibres and blood vessels. These last structures were NGF–immunoreactive only at early stages (foetal and P0).

**Conclusion:**

The results suggest that NGF signalling play an important role in the guidance of blood vessels and sympathetic fibres toward the islets during foetal and neonatal stages and could also preserve innervation at later stages of life.

## Background

Adult pancreatic β cells secrete insulin in response to an increase in extracellular glucose. At birth, this response is not fully developed as neonate β-cells are insensitive to glucose and synthesize and secrete less insulin than adults [1]. Pancreatic islets are innervated by autonomic fibres. In particular, sympathetic neural cell bodies are located in the superior mesenteric and celiac ganglia and are components of the splanchnic nerve and parasympathetic innervation comes from the vagus nerve [2].

Sympathetic innervation plays important roles in the physiology and physiopathology of the endocrine pancreas; nor- and epinephrine inhibit insulin secretion, while stimulate glucagon secretion [3]. Moreover, sympathetic innervation of pancreatic islets is altered in animal models with insulin resistance and type 2 diabetes [4]. However, factors that control sympathetic innervation of pancreatic islets are not clear. The neurotrophin nerve growth factor (NGF) is especially interesting because is synthesized by the peripheral targets of NGF-dependent sympathetic neurons and NGF concentrations are proportional to their innervation density [5]. For example, NGF participates in the innervation of spleen, peripheral lymph nodes and ovaries [6-8] and has also trophic effects on their vascularization [9].

Adult pancreatic beta cells produce and secrete NGF and express NGF high- (TrkA) and low-affinity (p75) receptors during lifespan and NGF is important for a normal islet morphogenesis during prenatal life [10,11] and have trophic effects on β cells survival, maturation and insulin secretion [1,12-14]. These effects are controlled by autocrine or paracrine mechanisms [15].

Sympathetic nerves in the pancreas are closely related to blood vessels [16], and nervous fibres are also in part guided by endothelium-derived signals [17]. Trophic interactions among nerve cells, blood vessels and islet cells are fundamental to assure an adequate pancreatic ontogeny and a mature assemblage and function.

NGF could be an important regulator molecule on innervation and vascularisation of the islets. The following observations indicate that this could be the case, 1) NGF overexpression in pancreatic β cells causes islet sympathetic hyper-innervation [18]. 2) When islet grafts to diabetic rats are pre-treated with NGF, sympathetic innervation is increased after transplantation [19]. 3) Pancreatic sympathetic innervation is reduced in NGF/Bax double knockout (KO) mice [20]. Finally, NGF *in vitro *induces development of glucose-induced insulin secretion in neonate rat β cells [14] and promotes neuron-like phenotypic changes in foetal and adult β cells [21]. The aim of this study was to investigate the morphophysiological relationships between innervation, vascularization and islet morphogenesis and function during ontogeny of rat pancreas, in particular of islets from foetal (F19), early (P1) and late postnatal (P20) stages of development, compared to adults. We also analyzed biosynthesis and secretion of NGF and insulin by β cells at these stages.

## Results

### The structure of pancreatic islets during ontogeny

**Figure 1:**
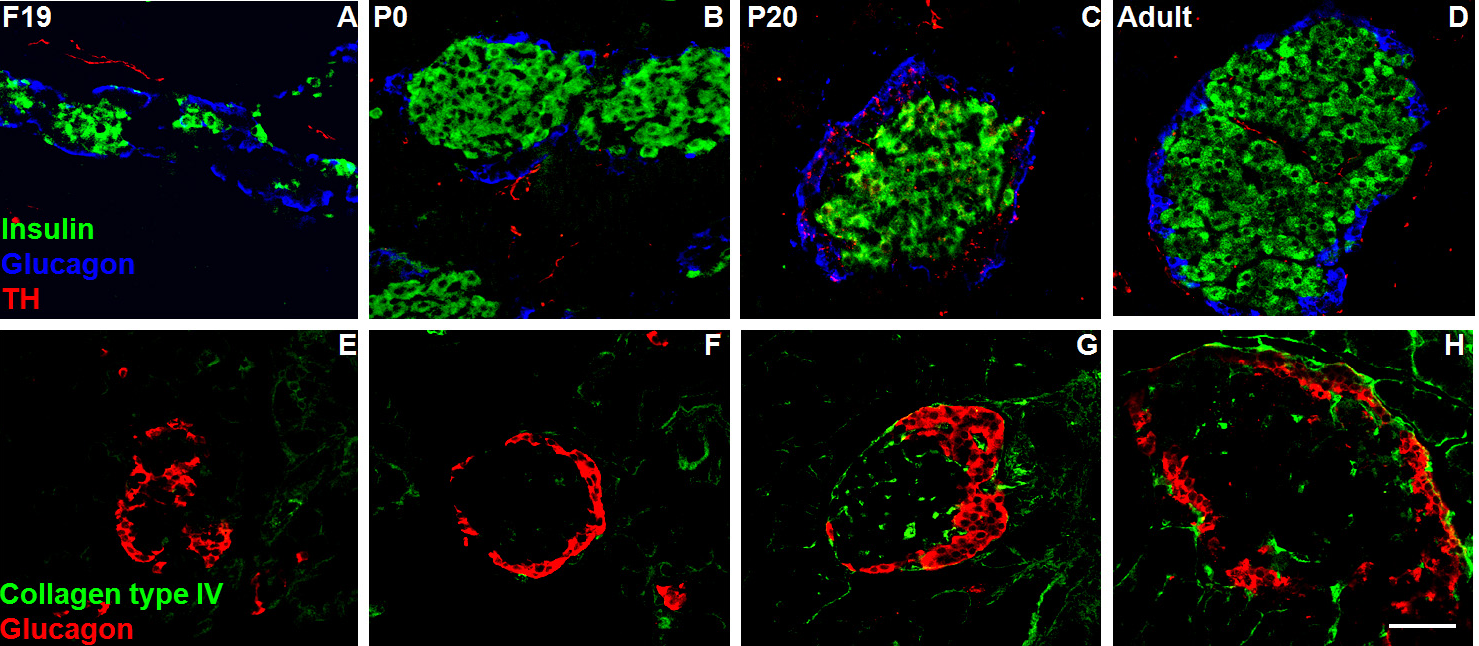
**Morphological reorganization of pancreatic islets from prenatal life to adulthood**. We localised sympathetic nerve fibres and collagen capsules surrounding the islets by confocal microscopy in longitudinal sections of pancreases. Immunostaining for insulin (green), glucagon (blue), tyrosine hydroxylase (TH; red) and collagen type IV (2^nd ^line, green) at different developmental stages (F19, P1, P20 and adult). Scale bar = 50 μm.

As expected, in mature adult islets glucagon producing β cells form a ring-like structure, surrounding insulin-producing β cells that fill most of the centre (Fig. 1D and 1H). At this stage, we observed that 98% of islets were delimited by a collagen capsule (Fig. 1H and additional file 1). In contrast, F19 islets were not formed yet; we only observed large groups of endocrine cells without a capsule and isolated α and β cells were scattered through the exocrine pancreas (Fig. 1A and 1E and additional file 1). Neonatal islets were small, rounded, did not yet presented capsule and were located in close proximity to each other (Fig. 1B and 1F and additional file 1). At P20 most of the endocrine cells had a similar distribution to adults; however, only nearly 26% of the islets were encapsulated (Fig. 1C and 1G and additional file 1).

### Remodelling sympathetic innervation of islets and NGF

**Figure 2:**
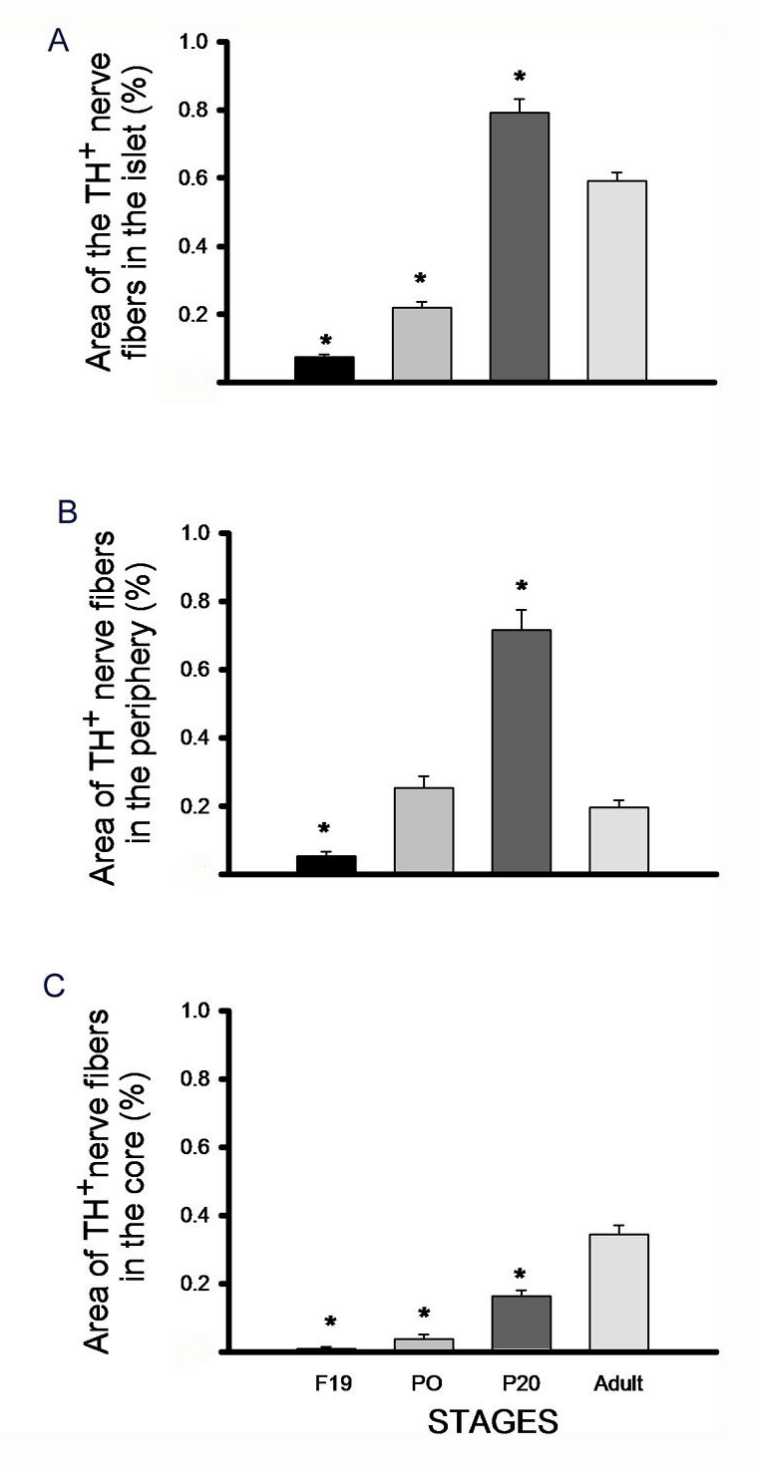
**Quantification and analysis of TH^+ ^fibres in development of islets**. Bar graphs represent A) Percentage of total area occupied by TH^+ ^fibres. B) Percentage of peripheral area occupied by TH^+ ^fibres. C) Percentage of the core area occupied by TH^+ ^fibres. ANOVA *p < 0.001 compared to adulthood.

To identify the sympathetic fibres we used a tyrosine-hydroxylase (TH) immunofluorescence. A progressive increase in the relative area occupied by TH positive innervation in the islets was observed between F19 to P20 (Fig. 1 upper panels and Fig 2A). Interestingly, this parameter decreased between P20 and adulthood in the periphery of the islets (Fig. 1 upper panels and Fig. 2B), while increased in the central area (Fig. 1, upper panels and Fig. 2C).

**Figure 3:**
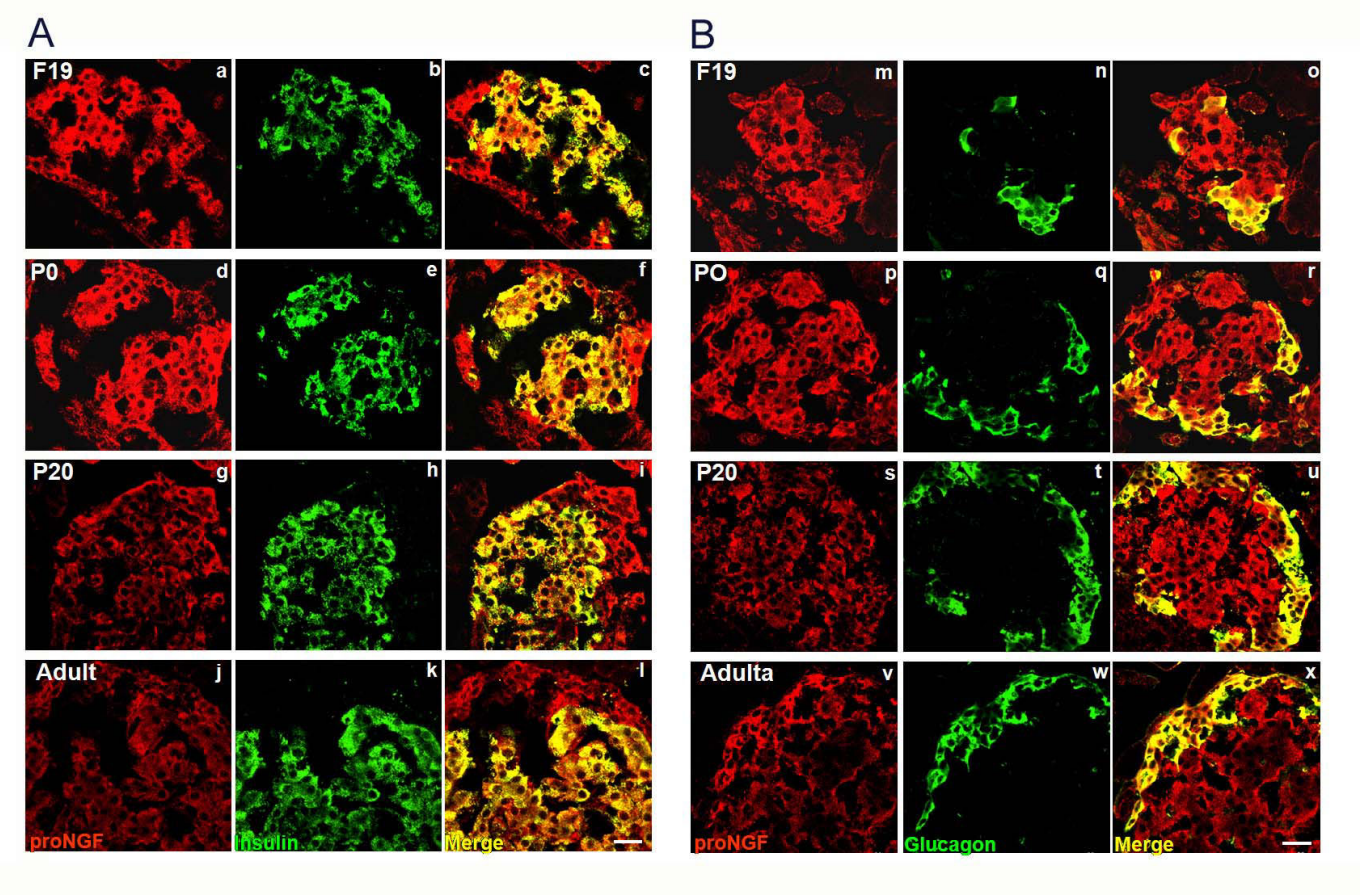
**Colocalization of pro-NGF and insulin or glucagon**. (A) Insulin and pro-NGF, (B) glucagon and pro-NGF at different developmental stages. Scale bar = 20 μm.

Sympathetic remodelling in this process is probably modulated by NGF produced by islet cells. We analyzed NGF production *in situ *with an antibody raised against the NGF precursor protein pro-NGF [22-24] in a and β cells. Pro-NGF immunoreactivity was observed in both cell types at all stages studied (Fig. 3A and 3B). However, the fluorescence intensity was higher at F19 and P1 than at later ontogenetic stages (additional file 2A and 2B) and when this was studied in isolated islet cells, we confirmed that most of them were β cells (insulin positive) and also labelled positive for TrkA receptors (additional file 3). At all stages studied mRNA for NGF is expressed by islet cells (data not shown).

### NGF is released by foetal and neonatal islet cells

**Figure 4:**
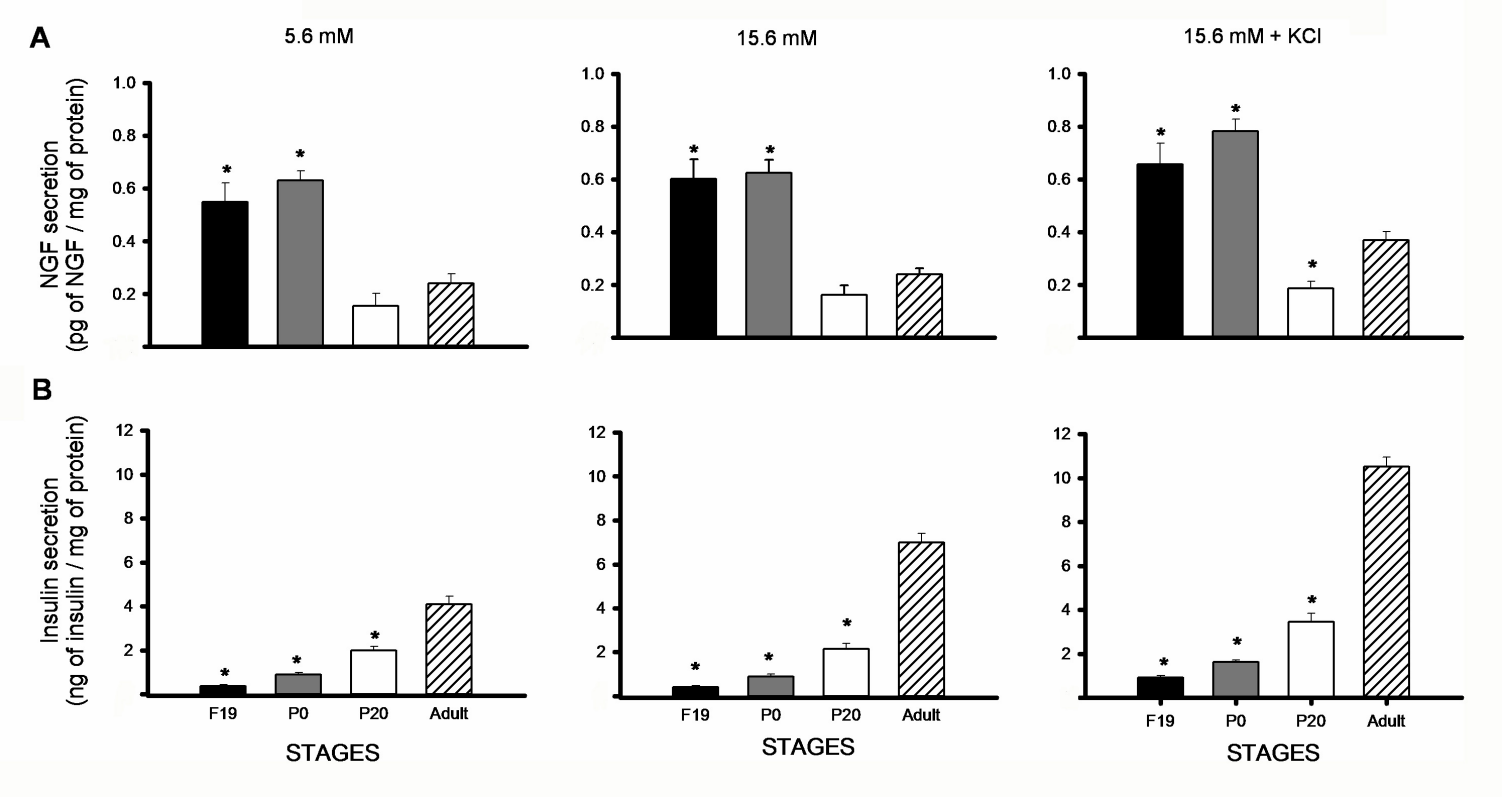
**NGF and insulin secretion at different stages of development**. Bar graphs represent NGF (A) or insulin (B) secretion by isolated insular cells under basal or stimulated conditions at different developmental stages. Line 1) Basal 5.6 mM glucose; 2) Stimulated 15.6 mM glucose and 3) Glucose 15.6 mM plus 20 mM KCl. ANOVA *p < 0.001 compared to adult cells. Data obtained from 4 independent experiments, each by duplicate.

We analyzed NGF secretion by ELISA (see Methods). At all studied stages, islet cells secreted NGF (Figure 4A). In accordance to the pro-NGF immunofluorescence observed; NGF secretion was higher at F19 and P1 than at older ages.

NGF secretion may be constitutive or regulated by other way than glucose stimulated insulin secretion, because clearly from figure 4B, both types of secretions are dissociated. This effect can not be attributed to cultured conditions or reduced cell viability, because P20 and adult cultured β cells increased insulin secretion when exposed to high glucose and KCl. Under the last experimental condition, adult β cells also increased NGF release.

### Pro-NGF and TrkA receptors are expressed for a delimited period in vascular cells

**Figure 5:**
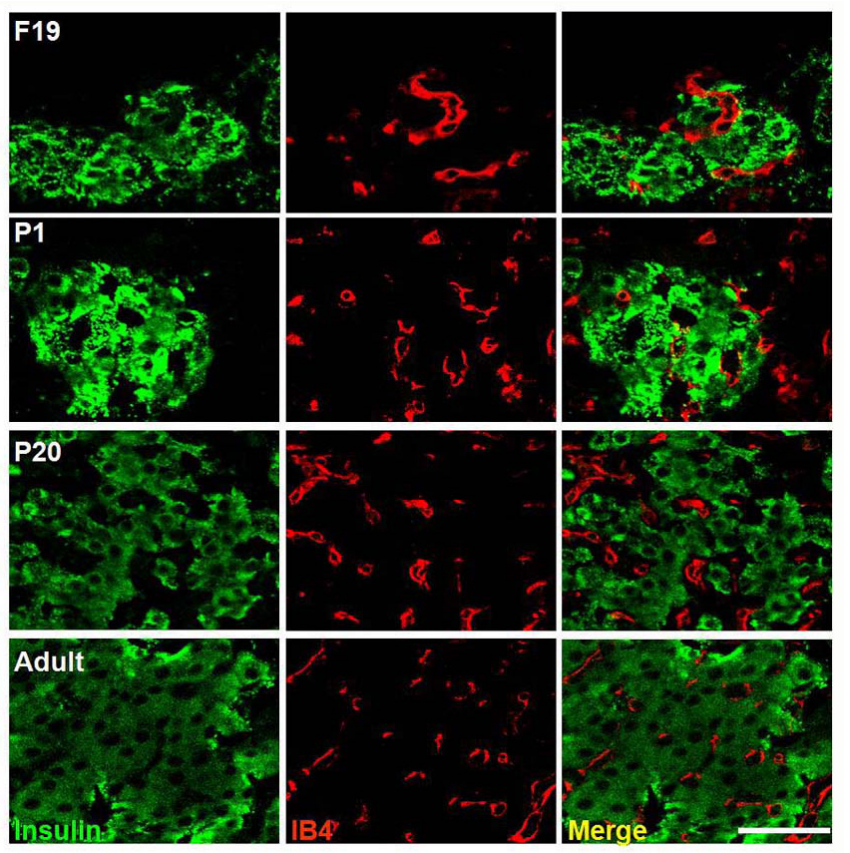
**Blood vessel localization in rat islets during development**. Insulin (green); Iso-lectin IB4 (red); merge (yellow). Scale bar = 50 μm.

**Figure 6:**
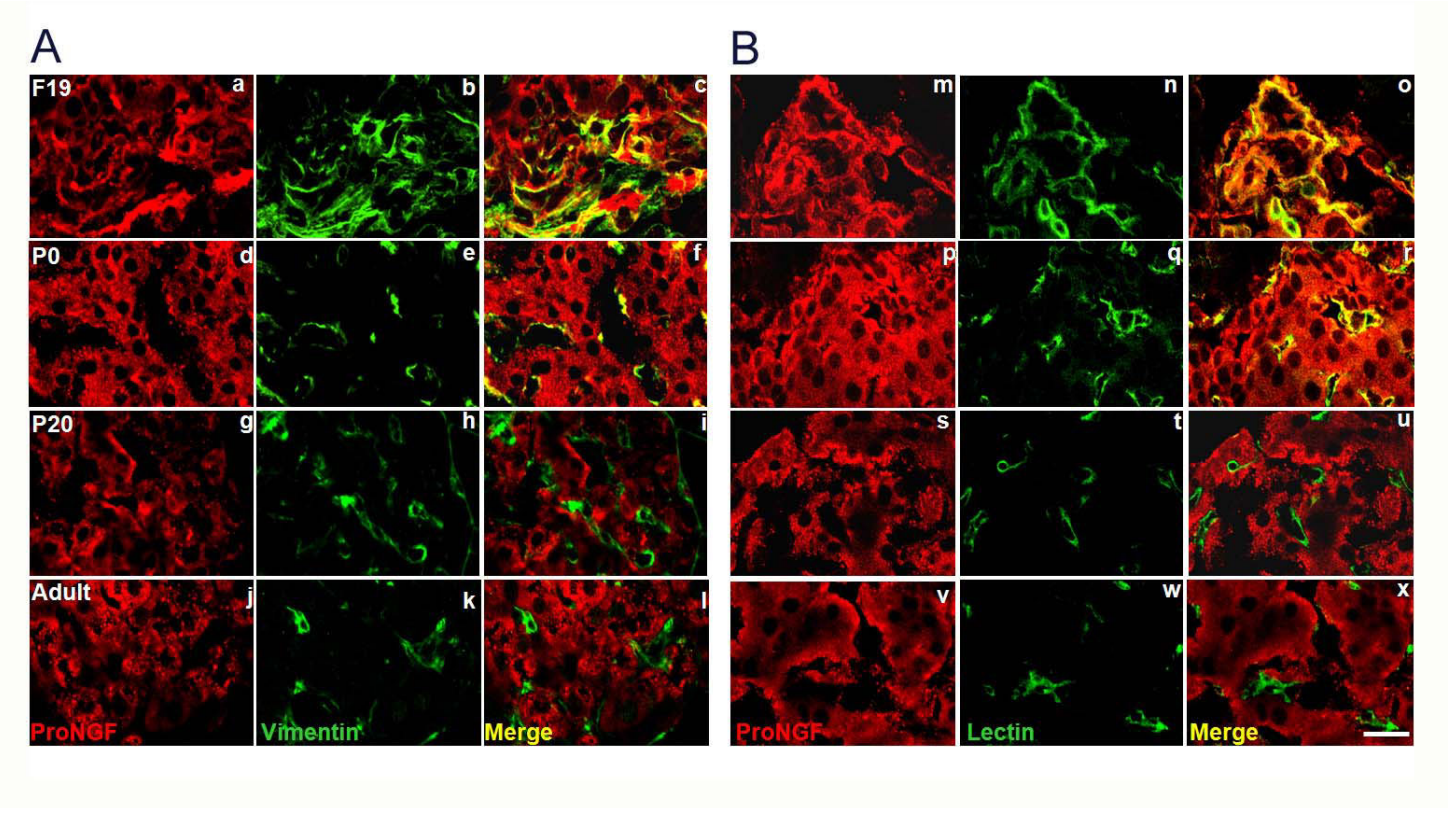
**Pro-NGF expression in vascular cells of pancreatic islets**. (A) Colocalization of pro-NGF and vimentin (c and f), (B) pro-NGF and lectin IB4 in different ontogenetic stages (o and r). Scale bar = 20 μm.

**Figure 7:**
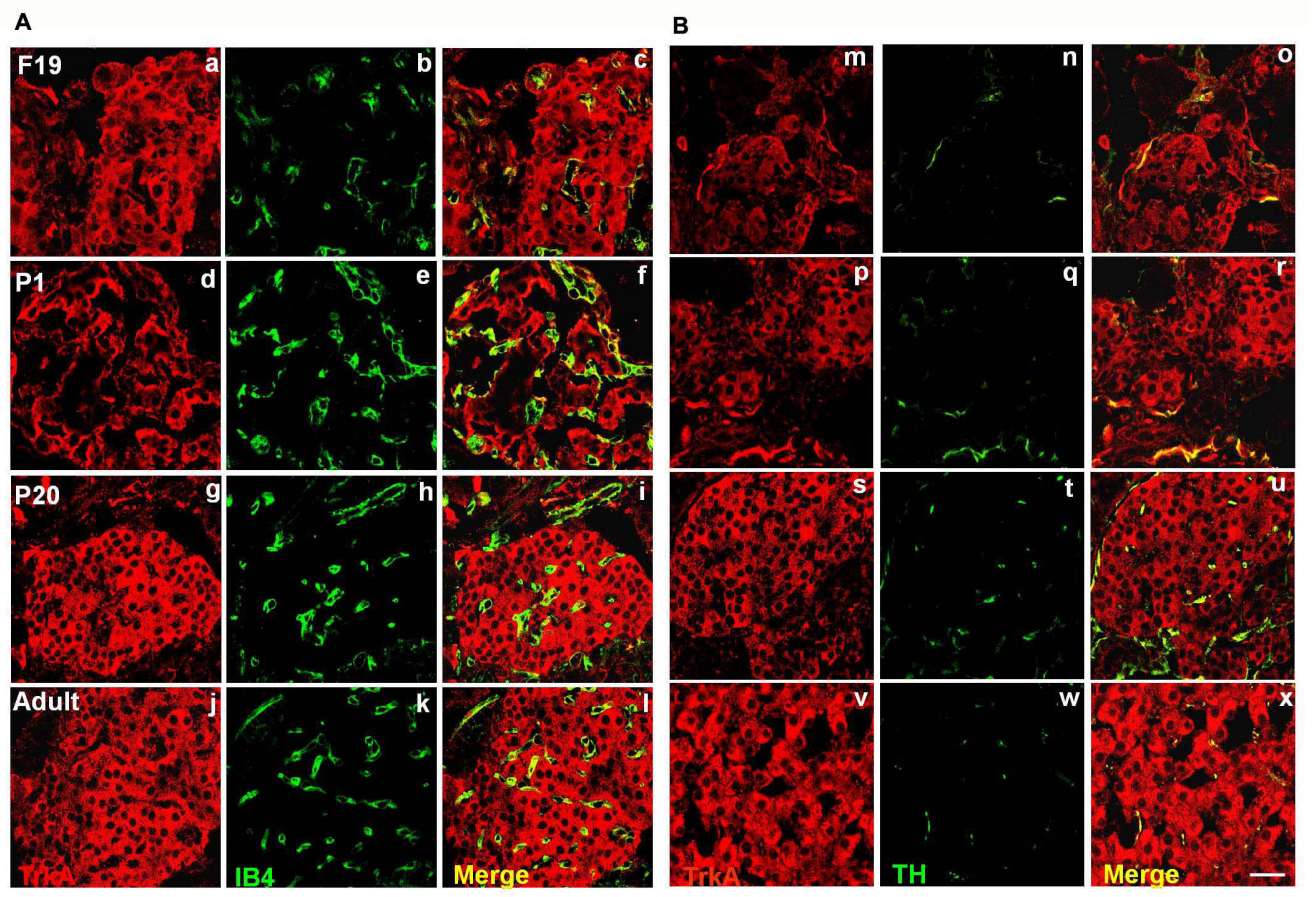
**Expression of TrkA in vascular cells and sympathetic fibres of pancreatic islets**. (A) Colocalization of TrkA and vimentin; (B) TrkA and TH at different ontogenetic stages. Colocalization of markers in blood vessels (c, f, i and l) or sympathetic fibres (o, r, u and x). Scale bar = 20 μm.

Vascular cells may also provide guidance cues to axons in their way to reach their targets [17]. Figure 5 shows that blood vessels that enter the islets early in pancreatic ontogeny (F19 and P1) labelled positive to pro-NGF, but not at P20 and thereafter (Fig. 6A and 6B). Blood vessels and sympathetic fibres can respond to NGF, because clear and consistently at all stages both vascular cells and TH positive fibres display immunoreactivity for TrkA receptors (Fig. 7A and 7B).

## Discussion

We analyzed innervation and function development of pancreatic islets. To our knowledge this is the first analysis of the relationship between innervation, blood vessels and rat pancreatic islets morphology at critical developmental stages. We also examined a possible role of NGF in this process.

Glucose homeostasis in foetal stages depends on the mother [25]. At birth, mammals abruptly need to achieve glucose control on their own, so β cells start secreting insulin. Nearly at P20 pups are weaned and they build up an omnivorous diet. These two stages are critical windows for pancreatic development and maturation of glucose induced insulin secretion [1,26]. We propose that a capsule or basal membrane surrounding the islets may be taken as one of the maturity markers, because almost all mature islets in adult animals are surrounded by one. It has been suggested that a membrane surrounding the islets could be an important niche for their complete development and survival [27-29]. We observed that a nearly-mature configuration of the pancreatic islets is progressively established during the first 20 days of posnatal life in the rat. However, at this stage nearly 30% of islets presented a continuous capsule surrounding them, indicating that they will continue maturing until adulthood [26].

We observed that pancreatic islets in early development receive an extensive innervation by sympathetic fibres. This innervation is refined later in development. Interestingly, when islets are transplanted to the kidney capsule or intraportally, they develop sympathetic innervation after transplantation [30,31]. Moreover, when purified β cells are transplanted to the kidney capsule, grafts are progressively innervated mainly by tyrosine hydroxylase-containing nerve fibres. These observations led to suggest that some factors produced by β cells mediate islet neurotrophism [31].

We have previously shown that adult pancreatic β cells produce and secrete NGF [15]. In this work we observed that this is the case also along ontogeny, which indicates that NGF may be trophic for sympathetic innervation and survival in islets, as it has been observed for other organs [6-8]. NGF secretion in adult cells could also be important for sympathetic fibres maintenance throughout life.

Vascular cells also displayed pro-NGF immunoreactivity, however this is only observed in a critical window around birth. This could also be important for nerve guidance into the islets. There is no perfect marker for blood vessels. We used isolectin IB4 and vimentin. Vimentin expression alone cannot be considered as specific endothelium marker; but it seems to accompany the process of angiogenesis in islets [32]. We also observed that both markers were expressed in blood vessels of developing and mature islets.

NGF produced by β, α and vascular cells might be acting by auto/paracrine mechanisms because all the studied cells within the islets express TrkA receptors. During development NGF may attract sympathetic fibres as they search for their targets as described for artemin [17,33] and/or promote sympathetic fibre outgrowth within the islet. The presence of TrkA receptors in β cells is not new [10,15,34]. It is likely that NGF acting through autocrine/paracrine mechanisms promotes β cell maturation. In support of this possibility, it has been shown that glucose-induced insulin secretion is increased by NGF after calcium channel translocation to the membrane of the neonate rat β cells [35]. The presence of TrkA receptors in vascular cells, on the other hand, might be important for islet blood vessel development because NGF also promotes angiogenesis [9,36] and migration of endothelial cell [37].

These results show that sympathetic innervation is subjected to a strong ontogenetic remodelling during pancreatic development. In general terms, there is a trend of innervation to increase from the perinatal period to P20. After this time, the amount of innervation within the islet decreases. Early hyper-innervation followed by a late elimination of redundant axons mediated through competitive interactions is commonly seen in peripheral targets [38,39]. However, it is to our knowledge the first description in pancreatic islets.

At earlier stages, sympathetic fibres are located mainly to the periphery of the islets or α cell compartment and as islets mature, an adjustment in the amount of fibres is accompanied by their relocation near the β cell area. Changes of preferred territories of axons to innervate during different ontogenetic stages are common. Transitory targets are used by growing axons while waiting for their definitive target to reach the maturity needed to receive innervation. This might happen in the pancreas since the innervation enters later to the β cell compartment, when these cells display more mature functional features [26]; in P28 through adulthood. Adult mice also show more sympathetic fibres located in the periphery of their islets [16].

An interesting issue that deserves consideration relates to the mechanism by which the sympathetic innervation is remodelled. Previous observations that show that NGF overexpression in transgenic mice [18] and knocking out NGF and BAX [20] led to hyper-innervation and hypo-innervation, respectively, strongly suggest that changes in NGF availability might underlie sympathetic nerve remodelling. Accordingly, our results show that insular cells synthesize and release higher amounts of NGF at F19 and P1 than at P20 and after. Interestingly, this pattern of NGF secretion closely paralleled the phases of hyper- and hypo-innervation described in this work.

Further support for this type of inference is the fact that TrkA-immunoreactivity was observed in islet sympathetic fibres at all ages. We may speculate that the decrease in NGF release in mature beta cells, might be the result of the negative feedback of norepinephrine, secreted by mature sympathetic fibres [40,41].

## Conclusion

**Figure 8:**
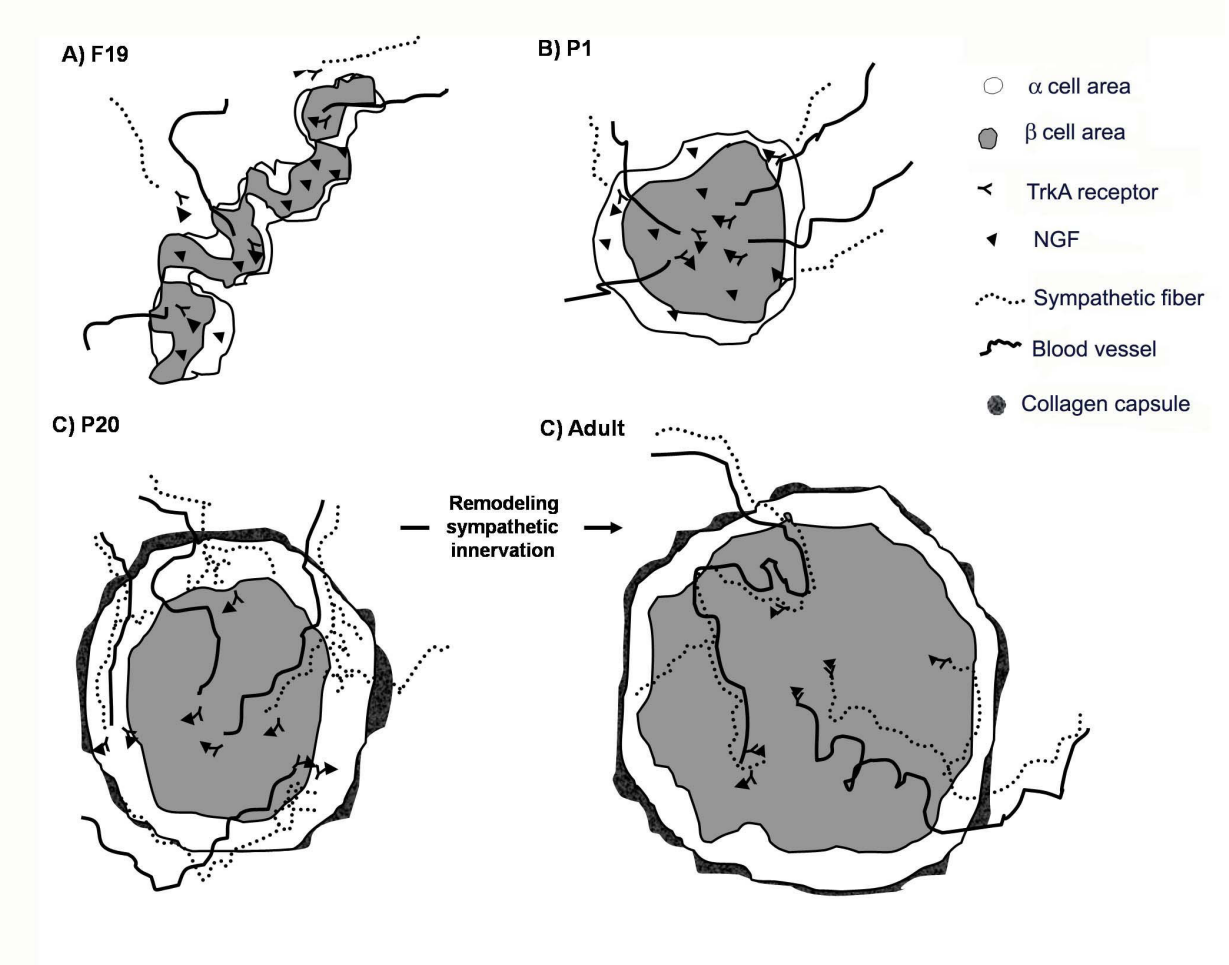
**Model to explain the possible role of NGF in pancreatic development**. Our results show that at foetal, early posnatal and P20 stages, the islets are immature in morphology and function. We observed that vasculature is formed first, followed by the innervation of sympathetic fibres and finally, by the capsule, finally conforming the architecture of the mature islet. Between P20 and adult stages a process of remodelling of the sympathetic innervation of the islet, probably by neural competition is observed. NFG could have a role in each the morphofunctional changes that can be received by insular cells, sympathetic fibres and blood vessels due to the presence to TrkA.

In conclusion, these morphological and functional data suggest that NGF plays a pivotal role in pancreatic islet morphogenesis, remodelling and maintenance of sympathetic innervation and development of the vasculature and maturation of a and β cells in the islets, as modelled in Fig 8.

There is increasing evidence supporting the causal connection between many adult diseases and an altered ontogeny caused by persistent interactions between the developing organism and its environment early during pre- or postnatal life. A decrease in islet innervation has been shown to be an early target in type 1 diabetes [42,43]. Moreover, both homozygous knockout mice for NGF and TrkA resulted in dramatic phenotypes showing poor viability, not surviving beyond few weeks [44].

Interestingly in humans, an autosomal recessive mutation in TrkA gene causes a syndrome of congenital insensitivity to pain with anhydrosis (CIPA; [45], reviewed in [46]). Patients with this mutation show an impaired first-phase of glucose induced-insulin secretion [47]. Finally, a decrease in serum NGF level has been associated with peripheral neuropathy in animal models and humans with diabetes [48,49].

Knowing the roles of NGF in pancreatic islet development, maturation, innervation and function may lead us to new perspectives for understanding and treatment of diabetes mellitus, including better conditions of islets for transplantation.

## Methods

### Animal care

The experiments were carried out in foetal (F19 day), neonate (1 postnatal day; P1), juvenile (P20) and adult Wistar rats. All postnatal rats were male, and born and raised in temperature and light controlled rooms located in our animal facility. Rats were weaned at P21 and allowed free access to food and water after weaning. Animal protocols were designed following the guidelines provided by the Guide for the Care and Use of Laboratory Animals (National Academy of Sciences, Washington D.C., 1996) and approved by the Animal Care Committee at the Instituto de Fisiología Celular, Universidad Nacional Autónoma de México.

### Culture of single insular cells

Rat foetuses were obtained through a caesarean incision at the age 19 days of gestation. In all the experiments, animals were deeply anesthetised with sodium pentobarbital (40 mg/kg of body weight). After this procedure, foetuses were removed and placed in culture dishes filled with cold D-MEM and rapidly, pancreas was dissected and animals were killed by cervical dislocation.

A similar protocol was followed in rats of different postnatal ages. Once pancreatic islets were obtained by collagenase digestion (collagenase type IV from Worthington; Freehold, NJ), insular cells were desegregated and cultured as described previously [13]. To improve dissociation, the suspension was passed through syringes with needles of decreasing diameter. Dissociated cells were cultured in 24-well culture dishes filled with RPMI 1640 (11.6 mmol/l glucose) supplemented with 10% foetal calf serum, 200/ml penicillin G, 200 μg/ml streptomycin, and 0.5 μg/ml amphotericin B, at 37°C and 5% CO2 in air. Islet cells were allowed to recover by culturing them overnight before conducting further experiments. Reagents were obtained from the following: Bovine serum albumin (BSA), HEPES, poly-L-lysine, trypsin, from Sigma (St. Louis, MO); tissue culture dishes from Corning (Corning, NY); foetal bovine serum (FCS) from Equitech-BIO (Ingram, TX); Hanks' balanced salt solution (HBSS), RPMI 1640 salts, and penicillin-streptomycin-amphotericin B solution from Life Technologies (Grand Island, NY).

### Immunofluorescence techniques

Fresh body-tail segments of rat pancreas were rapidly placed in a buffered solution of paraformaldehyde (4%) overnight at 4°C, washed in phosphate buffer-saline (PBS; pH7.4, 0.1 M), and sequentially transferred to graded (10, 20 and 30%) solutions of sucrose in PBS for 24 h in each concentration. Tissue samples were then included in Tissue-Tek-II, cut longitudinally (5 or 10 μm) in a cryostat (Leica CM 1900) and mounted on to gelatin-coated slides. One tissue section was taken every 200 μm along each pancreas segment.

Tissue sections and cultured cells were first incubated with a blocking solution, with 2% bovine serum albumin (wt/vol) and 0.1% Triton X-100 (vol/vol) in PBS-t during 30 min at room temperature. Cell and tissue samples were then incubated with the primary polyclonal antibodies raised against various neural, vascular and pancreatic antigens and worked at different conditions (additional file 4); then washed and incubated with the corresponding secondary antibodies conjugated to various fluorophores (additional file 4). This allowed us to develop protocols for single, double or triple immunofluorescence labelling. After a final washing step with PBS, slides were cover-slipped with anti-fading agent (DAKO). Negative controls were designed as follows: pre-adsorption with the corresponding antigen (1 μg peptide/μg antibody) for the antibodies raised against NGF, insulin or glucagon. For the rest of the antibodies, we omitted primary or secondary antibodies.

To determine the percentage of cells positive to NGF, TrkA, insulin and glucagon, at least 1400 culture cells were counted per marker. Percentage of pancreatic islets that display complete capsules were obtained at different developmental stages, 100 islets were counted per stage.

### Endothelial lectin-binding

Endothelial labelling was also achieved by incubating the tissue sections with TRITC-conjugated Bandeiraea simplicifolia I isolectin B4 (IB4; 45 ug/ml; Sigma), diluted in PBS supplemented with 0.1 mg/ml CaCl2 and MgCl2 [22]. After an extensive wash, all of the slides were cover-slipped with anti-fading mounting medium.

To further characterise blood vessels we used vimentin that is an intermediate filament protein (IF). Vimentin is expressed in cells of mesenchymal origin, like are endothelial, fibroblasts and smooth muscle cells, all types present in blood vessels [23,24]. We used a monoclonal mouse Anti-Vimentin, Clone VIM 3B4, DAKO (see table S1) according to the manufacturer's instructions.

### Quantitative analysis of the sympathetic innervation

We measured islet innervation area at different stages, in 5 sections, taken each 100 μm in the pancreas, per animal (n = 8 animals per data). In each section, 15 different islets were measured. Each islet was delineated and TH+ cells were removed from images using a computer based imaging analysis system (Image J 1.36, Wayne Rasband; National Institutes of Health, Bethesda, MD). The percentage of total pancreatic area and α and β areas occupied by TH+ fibres were estimated. The total pancreatic area was calculated by dividing TH+ fibres area by total pancreatic islet area, and α and β cell areas. The rate area TH+ = area TH+ × 100/islet area (α cell area + β cell area).

### Confocal microscopy

Confocal images were obtained using a Fluoview FV1000 laser confocal system (Olympus) attached/interfaced to an Olympus IX81 inverted light microscope with a 40× glycerol-immersion objective and some images were obtained with zoom 2. We used an Olympus Fluoview FV1000 confocal microscope to observe the sections for Cy5 (excitation 647 nm/DF32, emission 680 nm/DF32), CY3 (excitation 550 nm nm/DF32, emission 570 nm/DF32) and FITC (excitation 488 nm/DF32, emission 522 nm/DF32).

For the semiquantitive immunoreactive pro-NGF analysis, all images were taken with the exact same confocal settings to obtain semi-quantitative fluorescent signal and the histograms. Semi-quantification of image intensities was done with Olympus FV10-ASW 1.4 software. Fluorescence measurements were performed automatically by the computer program over the selected areas of interest. The program measures the fluorescence signal of the cytoplasm of the insular cells and obtaining the mean fluorescence in function of the total number of pixels studied in the islets.

Insulin and NGF secretion assays. After obtaining the cells, 200 000 islet cells per well were incubated in Hanks' balanced salt solution HBSS (5.6 mM glucose, 0.1% BSA) for 1 hour at 37°C. Then, cells were clustered in three experimental groups and incubated, in fresh 5.6 mM glucose-HBSS, 15.6 mM glucose-HBSS or 15.6 mM glucose, 20 mM KCl in HBSS, for 1 hour at 37°C. At the end a protease inhibitors was added to each sample (Complete Mini protease inhibitor cocktail; Roche Molecular Biochemical, Mannheim, Germany). NGF and insulin concentrations were then measured by ELISA; EmaxImmunoassay System (Promega, Madison, WI) for NGF and Mercodia ultrasensitive rat insulin ELISA (ALPCO, Windham, NH) for insulin. NGF and insulin levels are reported in pg and ng respectively. All the results were obtained by duplicate and normalised per μg of total protein as estimated by using Bradford assays as recommended by the supplier (Bio-Rad kit 500-0002).

### Statistical analysis

All of the data are reported as means ± SEM; n denotes the number of cells/or experiments. Because the data distribution fitted a normal curve, we used a one-way ANOVA followed by a Bonferroni's test for multiple comparisons to carry out the statistical analyses (Stat view 4.57; Abacus Concepts, Cary, NC). The p value was set at 0.05.

## Abbreviations

**NGF**: nerve growth factor; **F19**: foetal day 19; **P0**: postnatal day 0 or neonatal; **P20**: postnatal day 20

## Authors' contributions

SCV carried out experiments, participated in design of the experiments, draft and final version of the manuscript. VNT carried out experiments of mRNA NGF RT-PCR, cultures and discussion of the manuscript. CSS carried out cultures and insulin secretion analysis, also discussed the manuscript. GGO participated in the design of experiments and quantification of innervation and in discussion and draft version. MH conceived the study, designed experiments, coordination of participants, writing the manuscript. All authors read and approved the final manuscript.

## Supplementary Material

Additional file 1 **Pancreatic islets that display complete capsules at different developmental stages.** Click here for file

Additional file 2 **Ontogeny Pro-NGF expression**. (A) Pro-NGF expression in the pancreas at different developmental stages. (B) Bars represent mean fluorescence intensity (arbitrary units). ANOVA * p = < 0.001 relative to adulthood, n = 8 different animals per developmental stage. Scale bar = 20 μm. Click here for file

Additional file 3 **Percentage of cells immunoreactive to NGF, TrkA, insulin and glucagon at F19.** Click here for file

Additional file 4 **Antibodies used in the experiments.** Click here for file
