# Supplementary material for: Remodelling sympathetic innervation in rat pancreatic islets ontogeny
Source: BMC Dev Biol. 2009 Jun 17;9:34. doi: 10.1186/1471-213X-9-34 (PMC2711085; doi:10.1186/1471-213X-9-34)
Supplement: Additional file 1 — Pancreatic islets that display complete capsules at different developmental stages. [file 1471-213X-9-34-S1.pdf]

ANTIBODY	SOURCE	CONDITIONS
1. Guinea pig anti-insulin	ICN, Aurora, OH	1:4000, 12 h, 4 °C (cells)<br>1:2000, 12 h, 4 °C (Tissue sections)
2. Mouse anti-glucagon	Sigma	1:12000, 12 h, 4 °C (cells)<br>1:6000, 12 h, 4 °C (Tissue sections)
3. Rabbit anti-TrkA	Abcam	1:100, 12 h, 4 °C (cells)<br>1:25, 12 h, 4 °C (Tissue sections)
4. Goat anti-glucagon	Santa Cruz	1:500, 12 h, 4 °C (Tissue sections)
5. Rabbit anti-NGF	US Biologycal	1:1000, 36 h, 4 °C (cells)
6. Rabbit anti-tyrosine hydroxylase	Chemicon	1:100, 36 h, 4 °C (Tissue sections)
7. Mouse anti-tyrosine hydroxylase	Chemicon	1:20, 36 h, 4 °C (Tissue sections)
8. Rabbit anti-ProNGF	Chemicon	1:50, 12 h, 4 °C (Tissue sections)
9. Mouse anti-vimentin	Dako	1:100, 12 h, 4 °C (Tissue sections)
10. Rabbit anti-GFP	Santa Cruz	1:200, 12 h, 4 °C (Tissue sections)
11. Mouse Anti-collagen type IV	Dako	1:100, 12 h, 4 °C (Tissue sections)
12. Cyanine 3 (Cy3)-conjugated F(ab')2 fragment of goat anti-guinea pig IgG	Jackson ImmunoResearch	1:50, 2 h, room temperature
13. Cyanine 5 (Cy5)-conjugated F(ab')2 fragment of goat anti-rabbit IgG	Zymed laboratories	1:100, 2 h, room temperature
14. Fluorescein isothiocyanate-conjugated (FITC)-conjugated anti-mouse IgG	Zymed laboratories	1:200, 1 h, room temperature
