# Supplementary figures and images for: Remodelling sympathetic innervation in rat pancreatic islets ontogeny
Source: BMC Dev Biol. 2009 Jun 17;9:34. doi: 10.1186/1471-213X-9-34 (PMC2711085; doi:10.1186/1471-213X-9-34)

**A**

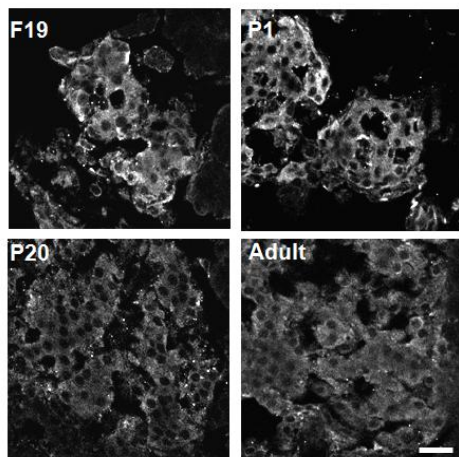

**B**

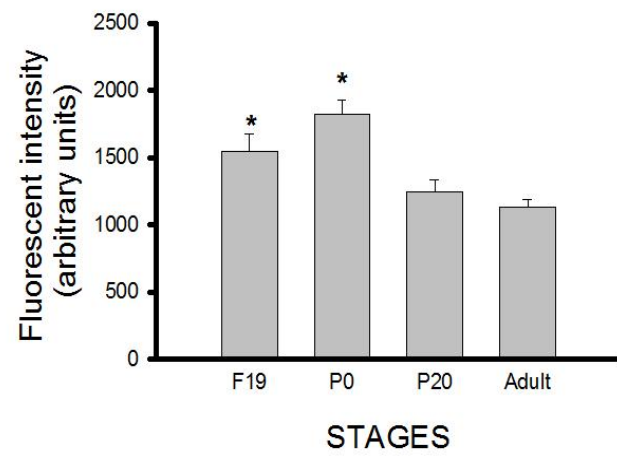

Supplement: Additional file 2 — Ontogeny Pro-NGF expression. (A) Pro-NGF expression in the pancreas at different developmental stages. (B) Bars represent mean fluorescence intensity (arbitrary units). ANOVA * p = < 0.001 relative to adulthood, n = 8 different animals per developmental stage. Scale bar = 20 μm. [file 1471-213X-9-34-S2.pdf]
