# Supplementary material for: Remodelling sympathetic innervation in rat pancreatic islets ontogeny
Source: BMC Dev Biol. 2009 Jun 17;9:34. doi: 10.1186/1471-213X-9-34 (PMC2711085; doi:10.1186/1471-213X-9-34)
Supplement: Additional file 3 — Percentage of cells immunoreactive to NGF, TrkA, insulin and glucagon at F19. [file 1471-213X-9-34-S3.pdf]

**STAGES**	**ENCAPSULATED ISLETS %**
F19	0
P1	0
P20	26
Adult	98
