# Supplementary material for: Remodelling sympathetic innervation in rat pancreatic islets ontogeny
Source: BMC Dev Biol. 2009 Jun 17;9:34. doi: 10.1186/1471-213X-9-34 (PMC2711085; doi:10.1186/1471-213X-9-34)
Supplement: Additional file 4 — Antibodies used in the experiments. [file 1471-213X-9-34-S4.pdf]

**Markers**	**Number of cells (%)**
NGF	87 ± 2.7
TrkA	82 ± 3.9
Insulin	74 ± 2.5
Glucagon	12 ± 1.8
